# A New Article in the Treatment of Gangrene

**Published:** 1847-02

**Authors:** 


*A new Article in the Treatment of Gangrene.*—To the Editor of the Boston Medical and Surgical Journal:—DEAR SIR,—I believe it to be a duty which I owe to science and humanity, to communicate to the profession, through your Journal, the result of some speculations and experiments I have been making in regard to an apparent specific in gangrene.

I have good reason to believe that *Sanguinaria*, given as a decoction, or in tincture, probably the former is to be preferred, will prevent and arrest mortification. That it will do so in cattle I am certain, and I have reasons to suppose that its effects upon the human subject would be no less beneficial.

As no surgeon can be expected to carry his love of experiments so far as to allow gangrene to commence for the purpose of arresting it by a new remedy, and as it may be difficult to say absolutely, that mortification would have taken place, where preventive measures have been seasonably prescribed; I cannot declare so positively as might be wished, that sanguinaria is a specific for gangrene; but I am so well satisfied of its good effects where a powerful antiseptic remedy has been indicated, that I should not feel justified in withholding my impressions from my professional brethren, nor, If I am not *too sanguine*, the benefits of such a discovery from the public.

When given given in tincture and decoction, the dose must of course be graduated to the strength of the patient, and so given as not to produce violent nausea or vomiting.

Dr. Mott is the only individual to whom I have communicated the above information. Very respectfully yours,

*New-York, Nov.* 28, 1846. H. BOSTWICK, M. D.

